# Incrementality in Planning of Speech During Speaking and Reading Aloud: Evidence from Eye-Tracking

**DOI:** 10.3389/fpsyg.2016.00033

**Published:** 2016-01-26

**Authors:** Lesya Y. Ganushchak, Yiya Chen

**Affiliations:** ^1^Leiden University Centre for Linguistics, Leiden UniversityLeiden, Netherlands; ^2^Education and Child Studies, Faculty of Social and Behavioral Sciences, Leiden UniversityLeiden, Netherlands; ^3^Leiden Institute for Brain and Cognition, Leiden UniversityLeiden, Netherlands

**Keywords:** sentence planning, discourse context, reading aloud, naming, eye-tracking, incrementality

## Abstract

Speaking is an incremental process where planning and articulation interleave. While incrementality has been studied in reading and online speech production separately, it has not been directly compared within one investigation. This study set out to compare the extent of planning incrementality in online sentence formulation versus reading aloud and how discourse context may constrain the planning scope of utterance preparation differently in these two modes of speech planning. Two eye-tracking experiments are reported: participants either described pictures of transitive events (Experiment 1) or read aloud the written descriptions of those events (Experiment 2). In both experiments, the information status of an object character was manipulated in the discourse preceding each picture or sentence. In the Literal condition, participants heard a story where object character was literally mentioned (e.g., *fly*). In the No Mention condition, stories did not literally mention nor prime the object character depicted on the picture or written in the sentence. The target response was expected to have the same structure and content in all conditions (*The frog catches the fly*). During naming, the results showed *shorter* speech onset latencies in the Literal condition than in the No Mention condition. However, no significant differences in gaze durations were found. In contrast, during reading, there were no significant differences in speech onset latencies but there were significantly *longer* gaze durations to the target picture/word in the Literal than in the No Mention condition. Our results shot that planning is more incremental during reading than during naming and that discourse context can be helpful during speaker but may hinder during reading aloud. Taken together our results suggest that on-line planning of response is affected by both linguistic and non-linguistic factors.

## Introduction

To produce a sentence, speakers must prepare a preverbal message and then encode it linguistically (e.g., lexical selection and phonological encoding; Levelt et al., 1999). Current theories of speech planning agree that speaking is an incremental process: speakers plan what they want to say in small chunks rather than planning a whole sentence (for review see Wheeldon, 2013). Thus, during speaking, planning and articulation overlap in time. More recently, Konopka and Meyer (2014) have also argued that during planning, the size of the planning unit may vary in different situations, resulting in a continuum of incrementality in planning (Konopka and Meyer, 2014). For instance, planning scope can be affected by the goal of the speaker (Ferreira and Swets, 2002), by language- specific linguistic features such as different phrasal word orders (Brown-Schmidt and Konopka, 2008), or even by the availability of cognitive resources (e.g., Wagner et al., 2010; Konopka, 2012). The goal of the present study is to investigate whether and how linguistic factors such as the information status of an event (i.e., given versus new) and non-linguistic factors such as the nature of the production task (i.e., picture naming versus reading aloud) affect the time course of on-line sentence formulation.

It is by now well-recognized that the process of planning for both picture naming and reading aloud is a highly dynamic one, a major reflection of which is the variability of the unit of planning within an utterance, ranging from an entire clause to a single phrase or a lexical item (for review see Konopka, 2012). Furthermore, zooming into the range of linguistic factors that affect planning, a consistent finding is that the accessibility or information status of the agent and patient of an event plays a significant role in the way utterances are formulated to describe the event. There is abundant evidence that speakers prefer to begin sentences with accessible characters (e.g., Prat-Sala and Branigan, 2000; Bock et al., 2004; Christianson and Ferreira, 2005; Branigan et al., 2008; Konopka and Meyer, 2014). So, easy-to-name characters become subjects more often than harder-to-name characters (e.g., Konopka and Meyer, 2014). This is in accordance with the so-called minimal load principle (Levelt, 1989) which states that completing easy processes before hard processes results in a lighter cognitive load on the production system, which in turn enables speakers to quickly begin and complete the encoding of individual increments (e.g., Ferreira and Henderson, 1998). For example, Konopka and Meyer (2014) showed that the planning of simple subject-verb-object (SVO) utterances was affected by the accessibility of a referent. Ganushchak et al. (2014) showed that information status (whether the information is new and therefor focused) also affects planning of utterances. In their experiments, Ganushchak et al. (2014) asked participants to describe pictures of two-character transitive events, while participants’ eye-movements were recorded. Discourse focus was manipulated by presenting questions before each picture. Their results showed that speakers rapidly directed their gaze to the *new* character they needed to encode.

Planning has also been reported to be affected by non-linguistic factors, such as the nature of the production task: reading versus. naming. Word reading and picture naming have been extensively studied throughout the history of psycholinguistics. Previous studies that explored word reading and picture naming in sentence context showed shorter latencies for word reading compared to latencies for picture naming (e.g., Potter et al., 1986; Theios and Amrhein, 1989). Furthermore, during scene description, utterance formulation begins with an apprehension phase (0–400 ms after picture onset) during which speakers encode the “gist” of the event. The apprehension phase is then followed by linguistic encoding that lasts until the end of articulation (Griffin and Bock, 2000). No such apprehension phase, however, is necessary during reading. Thus, picture naming requires conceptual preparation and selection of the correct name from other plausible alternatives, whereas reading could be achieved without access to the full semantic representation of the word (e.g., Potter et al., 1986; Levelt et al., 1999). There is evidence that during reading, the semantic system is recruited only when readers have difficulty to generate the pronunciation of a word by relying on orthography-to-phonology mapping alone (e.g., Cortese et al., 1997).

Thus far, the few studies that directly investigated the planning processes in reading versus naming have focused mainly upon the comparison between naming and reading of numerals (e.g., Ferrand, 1999; Meeuwissen et al., 2003; Korvorst et al., 2006). For instance, in an eye-tracking experiment, Korvorst et al. (2006) presented complex numerals in Arabic or an alphabetic format and asked participants to either name the numerals or read them aloud as house numbers or as clock times. They found that the degree of incrementality in planning was affected mainly by the nature of the utterance (house number versus clock times). Furthermore, utterance planning was influenced by different factors in the two production tasks but this was only evident in the production of clock times and not house numbers. Specifically, during the naming of clock times, gaze duration was affected by morpho-phonological (e.g., number of phonemes) as well as conceptual factors (e.g., factors related to telling time in Dutch; see Meeuwissen et al., 2003; Korvorst et al., 2006). However, during the reading of clock times, gaze durations reflected only morpho-phonological differences. This suggests that during reading aloud, conceptual preparation was no longer required (Meeuwissen et al., 2003; Korvorst et al., 2006). Thus, the presence and absence of conceptual preparation is responsible for the planning differences of an utterance between naming and reading tasks (Meeuwissen et al., 2003; Korvorst et al., 2006).

No study thus far, however, has investigated how linguistic (accessibility) and non-linguistic (production task) factors may interact to affect the planning of an utterance. To address this question, two comparable groups of participants were asked to describe a simple event (Experiment 1) or read aloud the written description of the same event (Experiment 2) while their eye-movements and onset speech latencies were recorded. Furthermore, we manipulated the accessibility of the object character of an event by providing two different discourse contexts prior to each picture or written sentence. In the *Literal condition*, the object character (e.g., *fly*) was literally mentioned in the preceding context. In the *No Mention condition*, stories did not literally mention nor prime any of the characters depicted on the picture. The target response was expected to have the same structure and content in all conditions (*The frog catches the fly*).

Differences in the planning of the target response in the naming and reading tasks were evaluated by the time needed for the preparation of speech, as reflected in speech onset time, but also by comparing speakers’ eye-movements to the object characters in the picture and object words in the written sentence, respectively. Gaze duration provides another good measure for estimating the total amount of speech planning that is required in order to produce an utterance (e.g., Meyer and Lethaus, 2004).

A distinction between early and late processing was also examined. Good index of early processing is (a) *first gaze durations*, which is the sum of all fixation durations on a target word/character prior to moving to another region. Measurement indexing the late processing is (b) *total gaze durations*, which is the sum of all fixations on a region. From previous literature, we know that first gaze duration is sensitive to earlier comprehension processes such as word recognition (see Clifton et al., 2007, for an overview), while total gaze duration reflects later processing such as re-analysis and discourse integration (e.g., Rayner, 1998; Frisson and Pickering, 1999; Sturt, 2007). Longer duration is usually taken as an indication of more effortful integration processes (e.g., Rayner and Sereno, 1994).

As mention above, in a picture description task, the formulation of a sentence begins with a short apprehension phase during which speakers encode the gist of the event (e.g., Griffin and Bock, 2000; Meyer and Lethaus, 2004; Konopka, 2014). Event apprehension is then followed by a longer phase of linguistic encoding. Typically, easy-to- name characters are fixated for less time than harder-to-name characters (e.g., Griffin and Bock, 2000; Meyer and Lethaus, 2004; Konopka, 2014). In a reading task, no apprehension phase is expected of the whole event described in the text. Readers typically do not need to read the whole sentence first, prior to start reading aloud. Another difference between reading and picture description is that during reading, a given word is likely to prompt the reader to try to integrate the word to the prior reference in the discourse context, whereas this integration process is likely to occur earlier (e.g., during the gist preparation stage) in the picture naming task.

Thus, we propose that during picture naming, the unit of planning is larger than during the reading aloud task. Speakers are unlikely to start speaking before they understood the gist of the event depicted in the picture. During reading, however, speakers start reading immediately after the onset of the written sentence. Therefore, we predict that first fixations to the object character will be earlier than speech onset in the naming task, but later than speech onset in the reading task. Consequently, the discourse context that we manipulated should also affect planning in naming and reading differently. Namely, the accessibility of an object character should ease the linguistic encoding phase in naming but not necessarily so in the reading task. We then predict that speakers will initiate their speech faster in the reading task than in the naming task. Furthermore, in the naming task, there should be faster onset latencies and shorter gaze durations in the Literal condition compared to the No Mention condition. In the reading task, however, no differences are expected in the onset latencies between the Literal and the No Mention condition. As for the eye gaze characteristics, we expect that the gaze duration to the object word should be less in the Literal condition than in the No Mention condition, as readers may recognize the target word from the preceding context, which in turn can facilitate the integration processes.

## Experiment 1. Planning of Speech During Speaking

### Methods

#### Participants

Thirty-one native Dutch speakers (28 women) participated in the experiment (mean age: 20 years; *SD* = 1.9 years). All participants were students of Dutch universities. The study was approved by the ethical committee board at Leiden University. Participants gave written informed consent prior to participating in the study and received course credits for their participation. Due to technical problem, data of one participant was excluded from the analysis.

#### Materials

**FIGURE 1:**
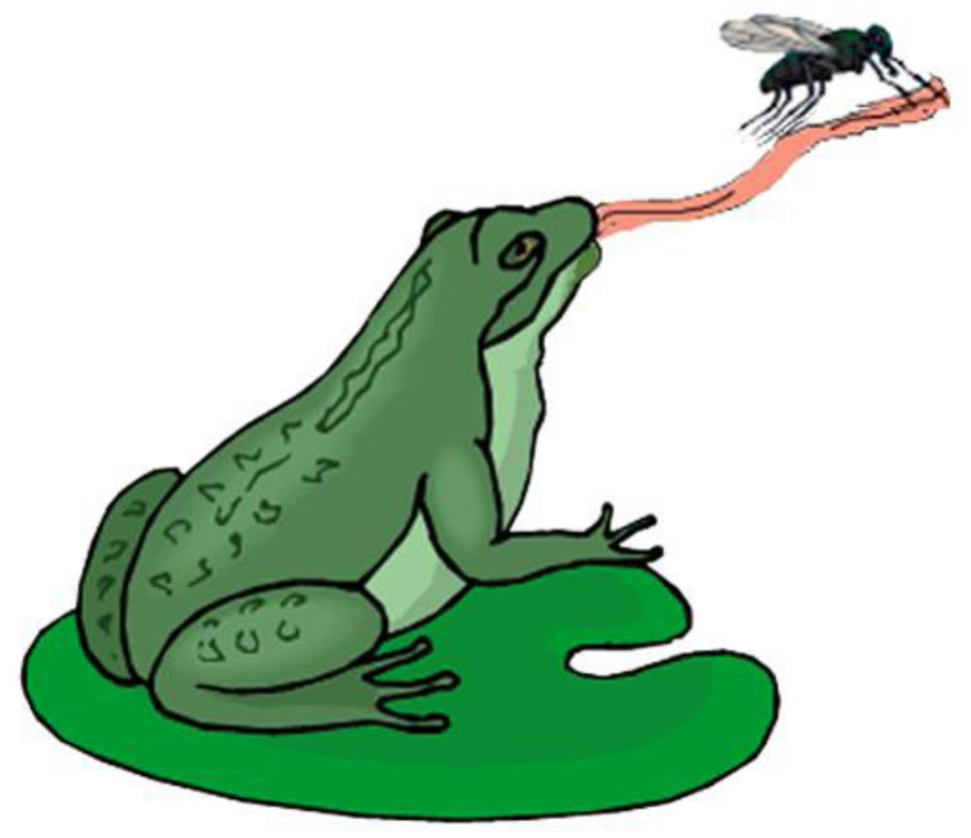
**Example of a target picture event**.

Seventy-eight colored pictures were used in the experiment (Konopka, 2014). All pictures displayed simple actions (**Figure 1**). There were 25 target pictures of transitive events, 50 fillers, and 3 practice pictures.

Accessibility was manipulated by means of short stories preceding each picture. All stories consisted of two sentences. The stories were only contextually related to the pictures, and were not intended to help participants understand the gist of the depicted event. Take the expected target sentence *De kikker vangt de vlieg* (‘The frog catches the fly’) as an example, the following illustrates the two conditions provided before the presentation of the target picture.

(1) Literal condition: The object character was literally mentioned in the preceding story. Note that the target object character was always placed in the same grammatical role as in the intended target sentence and it was always placed in the second sentence of the story.
*David vist regelmatig en weet dus ook het een en ander over vissen. Hij gebruikt een kleine vlieg als aas.* (David fishes regularly and knows a thing or two about fishing. He uses a small fly as bait.)
(2) No Mention condition: The story did not include literal or associative mention of words that describe characters in the picture.
*David gaat met zijn vader vissen. Ze gebruiken restjes van het avondeten als aas*. (David is going fishing with his father. They use leftovers from dinner as bait.)

All stories were pre-recorded by a native Dutch female speaker and presented auditorily prior to picture onset. For 40% of the filler trials, after the story, a yes-or-no comprehension question was presented visually on the computer screen. The purpose of the questions was to make sure that participants listened attentively to the presented stories.

#### Design and Procedure

Lists of stimuli were created to counterbalance story types across target pictures. Each target picture occurred in each condition on different lists, so that each participant saw each picture only once. Each subject saw eight target pictures per condition. There were at least two filler pictures separating any two target trials in each list.

**FIGURE 2:**
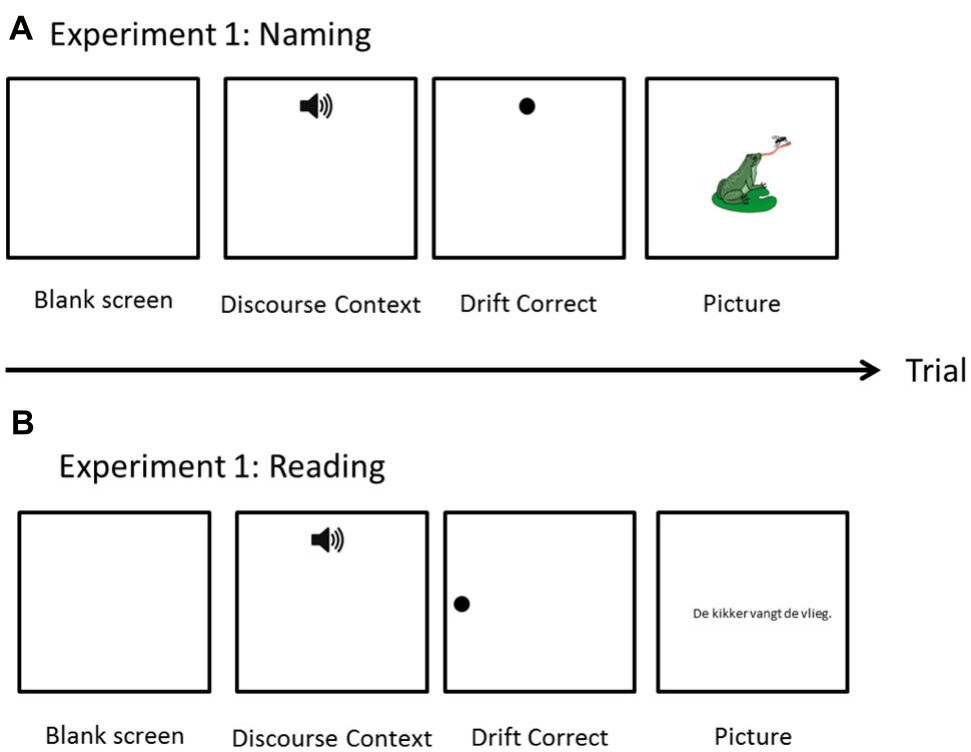
**Schematic representation of a trial for Naming (A) and Reading (B) experiments**.

Participants were seated in a sound-proof room. Eye movements were recorded with an Eyelink 1000 eye-tracker (SR Research Ltd.; 500 Hz sampling rate). Screen resolution was set at 1024 × 768. A 9-point calibration procedure was used. Eye calibration was done at the beginning of the experiment. The task started with three practice trials. Each trial started with the blank screen of 500 ms. followed, by the auditory presentation of the story (presented through headphones). The duration of the story varied (mean = 5804 ms; *SD* = 1302 ms). Simultaneously with the story, a pictorial representation of ‘Listen’ was presented at the top-center of the screen (as shown in **Figure 2**). On 40% of the filler items, there was a yes/no comprehension question presented prior to a picture trial. Participants used computer mouse to give their response. For all target trials and 60% of filler trials, after the completion of the story, the experiment proceeded to the picture trial. The picture trials began with drift correction, which also served as a fixation point, presented at the top of the screen. Afterwards, a picture was presented on the screen. Participants were instructed to describe each picture with one sentence which should mention all the characters in the picture. The time interval between offset of the auditory story and picture onset slightly varied per trials, as it was dependent on how quickly the eye fixations were registered during the drift correction phase. Participants were not under time pressure to produce the response. When the participant finished speaking, the experimenter clicked with the mouse to proceed to the next trial. On average, the pictures were displayed on the screen for 5227 ms (*SD* = 1604 ms).

#### Scoring and Data Analysis

Only responses with active SVO structure were scored as correct. Trials with a different structure (e.g., passive), wrong description, or corrections during the description were excluded from further analysis (Literal: 6.3%; No Mention: 7.2%).

Interest areas were drawn around each character in the target pictures (allowing a 2–3 cm margin around each character). Note, that the fixations were concentrated around the characters themselves; a more tightly fit ROI would not affect the reported results. Trials in which the first fixation was within the subject or object character interest area, instead of the fixation point, were also removed from further analyses (2% of the data). This left 440 trials for analysis. Analyses were carried out on speech onsets of correct responses. For the eye-tracking data, we determined first and total *gaze durations* for the targets. Speech onsets of correct responses and gaze durations were first log-transformed to remove the intrinsic positive skew and non-normality of the distribution (Baayen et al., 2008). Mixed-effects model analyses were carried out with participants and items as random effects and Condition (i.e., Literal and No Mention) as fixed effect. All models included random by-participant and by-item random intercepts and slopes for the factor Condition.

### Results

The time of first fixation on the subject character was on average 338 ms in the Literal condition and 336 ms in the No Mention Condition. Looks to the object character occurred at about 846 and 819 ms after the picture onset, in the Literal and No Mention condition, respectively. This is about 1000 ms *earlier* than the onset latencies (see **Table 1**). Note, the differences between times of the first fixation on the subject and object characters did not significantly differ across condition (all *t*s < 1.5).

**Table 1:** Mean response latencies in ms (and standard deviation) per condition in Naming (Experiment 1), in Reading (Experiment 2), and the mean difference across conditions (No Mention – Literal Mention).

	No Mention	Literal Mention	Mean Difference
Naming	1903 (496)	1841 (489)	62
Reading	834 (106)	827 (140)	7

#### Speech Onsets

Participants started speaking significantly earlier in the Literal compared to the No Mention condition (β = 0.05, *SE* = 0.02, *t* = 2.03, *p* = 0.04; see **Table 1**).

#### Eye-Tracking Data on Object Character

No significant difference was found in both the *first and total gaze durations* on the object character between the Literal and No Mention conditions (all *t*s < 1; see **Table 2** for means).

**Table 2:** Mean first and total gaze durations on object character/word in ms (and standard deviation) per condition in Naming (Experiment 1), in Reading (Experiment 2) and the mean difference across conditions (No Mention – Literal Mention).

	Naming		Reading	
	First gaze Duration	Total gaze Duration	First gaze Duration	Total gaze Duration
No Mention	558 (225)	1857 (440)	563 (155)	680 (175)
Literal Mention	542 (205)	1796 (527)	580 (160)	755 (221)
Mean Difference (No Mention – Literal	16	61	-17	-75

## Discussion

Overall, speech was initiated about 1000 ms after the first fixations to both the subject and object characters in the event pictures. This suggests that participants started to articulate the sentences after the apprehension phase and presumably after some of the linguistic encoding phase was completed. The onset of articulation was influenced by the discourse context manipulation. Speakers were significantly faster initiating production when the object character was given as compared to when the object character was contextually new. These results suggest that the activation of the object characters in the upcoming event facilitated planning. Note, that this does not necessarily mean that the speakers anticipated the upcoming events. Rather, we believe that it is likely due to the information about the object character given in the discourse context which made the encoding of the object character easier in the Literal condition than the No Mention condition. The lack of significant difference between the Literal and the No Mention condition in terms of both the *first and total gaze durations* on the object character suggest that neither object recognition nor integration into the discourse context were affected by our manipulation. We take this as evidence that the information status of the object character did not exert any effect on the planning of the initial chunk of speech in the naming task.

## Experiment 2. Planning of Speech During Reading Aloud

### Methods

#### Participants

Thirty-one native Dutch speakers (28 women) participated in the experiment (mean age: 20 years; *SD* = 1.9 years). None of the participants took part in Experiment 1. All participants were students of Dutch universities. The study was approved by the ethical committee board at Leiden University. Participants gave written informed consent prior to participating in the study and received course credits for their participation. Due to technical problem, data of one participant was excluded from the analysis.

#### Materials

The description of events produced by participants from Experiment 1 were used as targets in this experiment. To account for variability in responses, responses of each participant from Experiment 1 were used in its own list in the present study. Thus, 30 unique lists were created. Trials with erroneous responses were replaced by the corresponding standard target sentence (e.g., *De kikker vangt de vlieg*,‘The frog catches the fly’).

#### Design, Procedure, and Data Analysis

The design, procedure, and analyses were identical to Experiment 1. Interest areas were marked around target object words of each sentence as pre-defined by the analyzing software Data Viewer (SR Research Ltd.). Target trials with erroneous responses were removed from further analysis (Literal: 1.3%; No Mention: 3.0%). This left 472 trials for the analyses reported below.

### Results

The time of first fixation on the subject words was on average 293 ms in the Literal condition and 334 ms in the No Mention Condition. First fixation to the object words occurred at about 1221 and 1336 ms after the sentence onset in the Literal and No Mention condition, respectively. This is about 1000 ms *later* than speech onset (see **Table 1**). The difference between first fixation time between Literal and No Mention condition was not significant for looks to the subject word (*t* < 1). However, participants fixated on the object word significantly earlier in the Literal Mention than No Mention condition (β = 0.4, *SE* = 0.03, *t* = -2.40, *p* = 0.04)^1^.

#### Speech Onsets

No effects were found for speech onset latencies (all *t*s < 1; see **Table 1** for means).

#### Eye-Tracking Data on Object Word

No significant effects were found for *first gaze durations* (all *t*s < 1.5; see **Table 2** for means). However, there was a significant difference between conditions for *total gaze duration*. Namely, participants looked longer at the target word in the Literal condition compared to the No Mention condition (β = -0.2, *SE* = 0.06, *t* = -2.03, *p* = 0.04).

### Discussion

Contrary to Experiment 1, speakers initiated speech well before taking a look at the object word. This indicates that speakers started producing sentences before the comprehension of the whole event described in the written text. Onset latencies as well as first gaze durations were unaffected by the accessibility of the object character. However, total gaze durations were affected by the accessibility of the object character. Namely, in the Literal condition, participants looked at object character longer than in the No Mention condition.

## General Discussion

We reported two eye-tracking experiments that investigated the extent to which speakers’ simultaneous planning and articulation of an utterance is influenced by linguistic (accessibility) and non-linguistic (production task) factors. Our results show clearly that planning processes differ during naming and reading aloud. This is in accordance with previous findings (e.g., Meeuwissen et al., 2003; Korvorst et al., 2006). The crucial factor that influences planning in these two production tasks is conceptual preparation (Meeuwissen et al., 2003; Korvorst et al., 2006). In the naming task, participants had to describe events depicted on the pictures, which required conceptual preparation and selection of appropriate names for characters from the competing alternatives. In the reading task, however, no conceptual preparation and no word selection were necessary.

Another way to account for the differences between the naming and reading tasks is that in the picture naming task, the unit of planning was larger than during reading. In the picture description task, speakers initiated their speech around 1872 ms, much later than when they gazed upon the subject (337 ms after picture onset) and object characters (833 ms after picture onset). In the reading task, however, the initiation of speech was much earlier (830 ms), after they have looked at the subject word (312 ms after sentence onset), but much earlier than when they paid attention to the object word (1279 ms after sentence onset). This suggests that in naming, speakers had to encode the object character before they started speaking; while in the reading task, the object word is encoded only after the participants have already started articulating the first part of the sentence.

The differences in naming and reading aloud were also reflected in how discourse context affected the planning processes. In both speech production tasks, we observed effects of accessibility, which though manifested in two different ways. For the naming task, literal mention of the object character resulted in *facilitated* speech onset latency while no such effect was found in the reading task. This may be taken to indicate that the accessibility of the object did help to speed up the planning process during naming, probably all the way from the conceptualization of the message down to the retrieval and phonological encoding of the lexical item for the object. Our results do not allow to disentangle with certainty at which stage of planning (e.g., conceptual versus phonological) did the facilitation effect arise. In future studies, one might manipulate different levels of information that is provided by the discourse context (e.g., only conceptual information versus only phonological information).

In contrast, literal mention of the object character resulted in *inhibition* (as suggested by the longer gaze durations) during the reading task. Specifically, readers looked at the object word significantly longer in the Literal condition (755 ms) than in the No Mention condition (680 ms). Interestingly, the readers fixated on the object word significantly earlier in the Literal condition (1796 ms) than in the No Mention condition (1857 ms). These effects were not found for the naming task. Thus, it appears that readers look at the object word more quickly but also look at it for longer in the Literal condition than in the No Mention condition. The initial facilitation in the processing of the object word may come from the preview benefits from the parafoveal viewing. The preview benefits have been often demonstrated for the words that are orthographically or phonologically related to the target (for review see Schotter et al., 2012). There is also some evidence of processing of semantic information during the preview (e.g., Yan et al., 2009; Hohenstein et al., 2010). It is likely that the processing of object words was initially sped up by the orthographical and phonological (and possibly semantical) information that was activated by the discourse context. Note that the duration of the first fixation on a target word was slightly t shorter in the Literal condition (370 ms; *SD* = 139 ms) than in the No Mention condition (378 ms; *SD* = 120 ms), supporting the argument that word recognition processes might have benefited by the available information about the object word. The question that arises here is the later inhibition effects in the Literal Mention condition compared to the No Mention condition.

One reason could be that the inhibition effect resulted from competition of the phonology (and maybe orthography) of the previously activated word in the preceding discourse during the recognition of that same word in the reading of the post-discourse target sentence. Similar effects have been reported in Frisson et al. (2014), which though found an inhibition effect only when the two words overlapped in both phonology and orthography and were close to each other within one sentence. In our experiment, the effect, if verified to result from the same mechanism, was present even when they were as far apart as across different sentences.

Alternatively, this effect may be resulted from the fact that readers were trying to integrate the word to the prior reference in the preceding story. Two possible scenarios could have led to the observed gaze pattern. Possibility one is that such integration process might have been skipped or was shallow in the No Mention condition, compared to the Literal condition, since there was no obvious reference between the preceding context and the target sentence. Another possibility is that such an integration process turned out to be more costly when the given information of the object (provided in the discourse in the Literal condition) was coded with its full name as if it was new information. Further research is needed to find evidence for or against these speculations.

Taken together, our results show that planning is more incremental during reading, where planning and speaking are closely interleaved, than during naming. Reading tasks are often used to investigate language production processes. Our results show that nature of processes may differ across the tasks and that the time course of these processes may not be comparable for reading and naming tasks. Furthermore, our results showed that discourse context can be helpful during speaking but may hinder during reading aloud. Overall, our results suggest that planning is a dynamic process which is affected by both linguistic and non-linguistic factors.

## Author Contributions

LG: Substantial contributions to the conception or design of the work; the acquisition, analysis, and interpretation of data for the work; drafting the work and revising it critically; final approval of the version to be published; agreement to be accountable for all aspects of the work in ensuring that questions related to the accuracy or integrity of any part of the work are appropriately investigated and resolved. YC: Substantial contributions to the conception or design of the work; interpretation of data for the work; critically revising the manuscript; final approval of the version to be published; agreement to be accountable for all aspects of the work in ensuring that questions related to the accuracy or integrity of any part of the work are appropriately investigated and resolved.

## Conflict of Interest Statement

The authors declare that the research was conducted in the absence of any commercial or financial relationships that could be construed as a potential conflict of interest.

## Footnotes

1 Note, that the sentence length and number of words prior to the object word is comparable for the Literal (5.19 words; SD = 0.38 words) No Mention conditions (5.22 words; SD = 0.25 words).

## References

[B1] Baayen R. H. Davidson D. J. Bates D. M. (2008). Mixed-effects modeling with crossed random effects for subjects and items. *J. Mem. Lang.* 59 390–412. 10.1016/j.jml.2007.12.005

[B2] Bock K. Irwin D. E. Davidson D. (2004). “Putting first things first,” in *The Integration of Language, Vision, and Action: Eye Movements and the Visual World*, eds Ferreira F. Henderson M. (New York, NY: Psychology Press), 249–278.

[B3] Branigan H. P. Pickering M. J. Tanaka M. (2008). Contributions of animacy to grammatical function assignment and word order during production. *Lingua* 118 172–189. 10.1016/j.lingua.2007.02.003

[B4] Brown-Schmidt S. Konopka A. (2008). Little houses and casas pequenas: message formulation and syntactic form in unscripted speech with speakers of English and Spanish. *Cognition* 109 274–280. 10.1016/j.cognition.2008.07.011 18842259 PMC2665878

[B5] Christianson K. Ferreira F. (2005). Conceptual accessibility and sentence production in a free word order language (Odawa). *Cognition* 98 105–135. 10.1016/j.cognition.2004.10.006 16307955

[B6] Clifton C. Jr. Staub A. Rayner K. (2007). “Eye movements in reading words and sentences,” in *Eye Movements: a Window on Mind and Brain*, eds Gompel R. P. G. van Fischer M. H. Murray W. S. Hill R. L. (Oxford: Elsevier), 341–373.

[B7] Cortese M. J. Simpson G. B. Woolsey S. (1997). Effects of association and imageability on phonological mapping. *Psychon. Bull. Rev.* 4 226–231. 10.3758/BF03209397 21331829

[B8] Ferrand L. (1999). Why naming takes longer than reading? The special case of Arabic numbers. *Acta Psychol.* 100 253–266. 10.1080/0361073X.2011.568805

[B9] Ferreira F. Henderson J. M. (1998). Linearization strategies during language production. *Mem. Cogn.* 26 88–96. 10.3758/BF03211372 9519699

[B10] Ferreira F. Swets B. (2002). How incremental is language production? Evidence from the production of utterances requiring the computation of arithmetic sums. *J. Mem. Lang.* 46 57–84. 10.1006/jmla.2001.2797

[B11] Frisson S. Koole H. Hughes L. Olson A. Wheeldon L. (2014). Competition between orthographically and phonologically similar words during sentence reading: evidence from eye movements. *J. Mem. Lang.* 73 148–173. 10.1016/j.jml.2014.03.004

[B12] Frisson S. Pickering M. J. (1999). The processing of metonymy: evidence from eye movements. *J. Exp. Psychol.* 25 1366–1383. 10.1037//0278-7393.25.6.1366 10605827

[B13] Ganushchak L. Y. Konopka A. E. Chen Y. (2014). Focus planning during sentence production: an eye-tracking study. *Poster Presented on International Seminar on Speech Production (ISSP)*, Cologne.

[B14] Griffin Z. M. Bock J. K. (2000). What the eyes say about speaking. *Psychol. Sci.* 11 274–279. 10.1111/1467-9280.00255 11273384 PMC5536117

[B15] Hohenstein S. Laubrock J. Kliegl R. (2010). Semantic preview benefit in eye movements during reading: a parafoveal fast-priming study. *J. Exp. Psychol. Learn. Mem. Cogn.* 36:1150 10.1037/a0020233 20804291

[B16] Konopka A. E. (2012). Planning ahead: how recent experience with structures and words changes the scope of linguistic planning. *J. Mem. Lang.* 66 143–162. 10.1016/j.jml.2011.08.003

[B17] Konopka A. E. (2014). Speaking in context: discourse influences formulation of simple sentences. *Poster presented at the 27th CUNY Human Sentence Processing Conference*, Columbus, OH.

[B18] Konopka A. E. Meyer A. S. (2014). Priming sentence planning. *Cogn. Psychol.* 73 1–40. 10.1016/j.cogpsych.2014.04.001 24838190

[B19] Korvorst M. Roelofs A. Levelt W. J. M. (2006). Incrementality in naming and reading complex numerals: evidence from eyetracking. *Q. J. Exp. Psychol.* 59 296–311. 10.1080/17470210500151691 16618635

[B20] Levelt W. J. M. (1989). *Speaking: From Intention to Articulation*. Cambridge, MA: MIT Press.

[B21] Levelt W. J. M. Roelofs A. Meyer A. S. (1999). A theory of lexical access in speech production. *Behav. Brain Sci.* 22 1–38. 10.1017/S0140525X99001776 11301520

[B22] Meeuwissen M. Roelofs A. Levelt W. J. M. (2003). Planning levels in naming and reading complex numerals. *Mem. Cogn.* 31 1238–1248. 10.3758/BF03195807 15058685

[B23] Meyer A. S. Lethaus F. (2004). “The use of eye tracking in studies of sentence generation,” in *The Interface of Language, Vision, and Action: Eye Movements and the Visual World*, eds Henderson J. M. Ferreira F. (New York, NY: Psychology Press), 191–212.

[B24] Potter M. Kroll J. Yachzel B. Carpenter E. Sherman J. (1986). Pictures in sentences: understanding without words. *J. Exp. Psychol.* 115 281–294. 10.1037/0096-3445.115.3.281 2944988

[B25] Prat-Sala M. Branigan H. P. (2000). Discourse constraints on syntactic processing in language production: a cross-linguistic study in English and Spanish. *J. Mem. Lang.* 42 168–182. 10.1006/jmla.1999.2668

[B26] Rayner K. (1998). Eye movement in reading and information processing: 20 years of research. *Psychol. Bull.* 124 372–422. 10.1037/0033-2909.124.3.372 9849112

[B27] Rayner K. Sereno S. C. (1994). “Eye movements in reading: psycholinguistic studies,” in *Handbook of Psycholinguistics*, ed. Gernsbacher M. A. (San Diego, CA: Academic Press), 57–81.

[B28] Schotter E. R. Angele B. Rayner K. (2012). Parafoveal processing in reading. *Attent. Percept. Psychophys.* 74 5–35. 10.3758/s13414-011-0219-2 22042596

[B29] Sturt P. (2007). Semantic re-interpretation and garden path recovery. *Cognition* 105 477–488. 10.1016/j.cognition.2006.10.009 17178115

[B30] Theios J. Amrhein P. C. (1989). Theoretical analysis of the cognitive processing of lexical and pictorial stimuli: reading, naming, and visual and conceptual comparisons. *Psychol. Rev.* 96 5–24. 10.1037/0033-295X.96.1.5 2928419

[B31] Wagner V. Jescheniak J. D. Schriefers H. (2010). On the flexibility of grammatical advance planning during sentence production: effects of cognitive load on multiple lexical access. *J. Exp. Psychol.* 36 423–440. 10.1037/a0018619 20192540

[B32] Wheeldon L. (2013). “Producing spoken sentences: the scope of incremental planning,” in *Cognitive and Physical Models of Speech Production, Speech Perception, and Production-Perception Integration*, eds Perrier P. Lang P. (Berlin: Springer-Verlag GmbH).

[B33] Yan M. Richter E. M. Shu H. Kliegl R. (2009). Readers of Chinese extract semantic information from parafoveal words. *Psychon. Bull. Rev.* 16 561–566. 10.3758/PBR.16.3.561 19451385

